# Marginal and Para-Marginal Technique in Late Germectomy of Lower Third Molars

**DOI:** 10.3390/children10061077

**Published:** 2023-06-19

**Authors:** Francesco Saverio Ludovichetti, Sergio Mazzoleni, Manuel Menin, Roberta Gaia Parcianello, Francielle Romanowski, Edoardo Stellini, Andrea Zuccon

**Affiliations:** 1 Department of Neurosciences—Dentistry Section, Padova University, 35128 Padova, Italy; sergio.mazzoleni@unipd.it (S.M.); manuel.menin@studenti.unipd.it (M.M.); edoardo.stellini@unipd.it (E.S.); andrea.zuccon@unipd.it (A.Z.); 2 Department of Pediatric Dentistry, Milano University, 20100 Milano, Italy; robertagaia.parcianello@gmail.com; 3 Department of Pediatric Dentistry, Unievangelica University, Anapolis 75083-515, Brazil; francielleromanowski@hotmail.com

**Keywords:** germectomy, marginal flap, para-marginal flap, third molars, children

## Abstract

Introduction: Third molar surgery can cause post-operative complications to the patient due to the presence of a wound from the incision. This study aimed to compare marginal flap with para-marginal flap on postoperative complications by the measurements on pre and post-surgical plaque index (PI), bleeding on probing (BoP), maximum opening of the mouth (MOM), pain perception (PP) and post-surgical tumefaction (PT). Methods: In this double-blind randomized clinical trial, 40 patients were recruited and randomly allocated in two groups. In Group 1, third molar extraction was carried out after a marginal flap design was performed, while in Group 2 a para-marginal flap was performed. Plaque index, bleeding on probing, maximum mouth opening, and distal probing were assessed right before and one week after surgery, while post-operative pain perception and post-operative swelling were accounted one week following surgery. Results: Statistically significant differences were reported between treatment groups, as the para-marginal flap led to better outcomes for all the measured indexes. Conclusions: The para-marginal flap design may decrease the occurrence of post-operative complications and discomforts after mandibular third molar surgery, compared to marginal flap design.

## 1. Introduction

Third molars, commonly referred to as wisdom teeth, are situated in the posterior region of the oral cavity. They are the final permanent teeth to erupt and exhibit the greatest degree of variability in terms of calcification and eruption among all tooth types. This variability is not directly associated with gender, sexual maturation, or growth, as observed in other permanent teeth. However, certain differences can be attributed to ethnicity [1].

The crown of the wisdom tooth initiates its formation with the development of the primitive dental lamina between the ages of 4 and 5. Mineralization commences between the ages of 9 and 10, and crown formation is typically completed between the ages of 12 and 15. Subsequently, the eruptive phase ensues, facilitated by an ascending movement. The inclination of the tooth axis is primarily influenced by the available space in the retro-molar trigone and the growth of the posterior arch. Third molars (LM3) generally achieve full development between the ages of 18 and 24 before becoming visible [2,3].

While impacted or partially erupted wisdom teeth often do not present problems, some individuals may experience pain, swelling, or pericoronitis. Wisdom teeth can also cause damage to neighboring teeth, increase the risk of tooth decay, periodontal probing, root resorption, and the development of odontogenic cysts or tumors at the level of the second lower molar (LM2) [4,5].

Orthodontic complications, such as insufficient space for third molar eruption, impaction of the lower second molar, and impairment of second molar distalization in Class III malocclusions, may also arise. Historically, these complications were the primary reasons for the nearly universal extraction of third molars. However, contemporary dentistry exhibits increased caution towards their extraction due to the potential for additional complications. Consequently, careful consideration is necessary when determining the indication for their removal, with a thorough evaluation through orthopantomography and CBCT imaging. Common complications associated with wisdom tooth extraction include swelling, pain, secondary infection, trismus, ecchymosis, hemorrhage, and alveolar osteitis. Although inferior alveolar or lingual nerve paresthesia can occur, fortunately, they are uncommon. To avoid the aforementioned clinical issues, an increasing number of oral surgeons are inclined to perform germectomy in young patients, as a less invasive alternative to a potentially more invasive and higher-risk surgical procedure [6,7,8].

Germectomies can be performed at various stages of third molar development. Very early germectomies are typically conducted between 7 and 11 years of age, when the bony crypt of the third molar is well-defined and usually located near the anterior border of the ramus. This approach allows for a shorter and less invasive treatment, often concluding with simple curettage or direct aspiration of the dental germ. Early germectomies are carried out between 12 and 15 years of age when the roof of the bone crypt is not fenestrated. At this stage, it becomes necessary to surgically open the crypt, presenting a challenging procedure as the tooth germ tends to pivot easily on itself, requiring crown sectioning. The most favorable stage for germectomies is considered between 14 and 18 years of age, as the crypt’s bony cover is partially resorbed, while the crown remains submucosal. Despite being enclosed in its follicular membrane, the tooth is not at risk of infection. If extraction becomes necessary, it is best to perform the operation before the crown emerges [9,10].

In general, the extraction of impacted wisdom teeth and germectomies involve incising soft tissues, detaching the periodontal ligament if present, removing bone with high-speed drills and bone-cutting burs, and sometimes performing tooth separation if necessary. Following these steps, bruising of the alveolar site and irrigation with chlorhexidine or physiological solution are recommended for thorough debris removal. Finally, 4/0 sutures are applied to ensure proper wound healing. Unfortunately, these intervention steps can lead to various aforementioned side effects. Thus, the choice of flap design and type of surgical bone removal are crucial for achieving safe and satisfactory results. However, some research findings indicate no significant differences between flap types in terms of post-operative clinical attachment level, probing depth on the LM2, swelling, or post-operative pain perception. Conversely, the choice of incision type may influence maximum mouth opening [11,12]. Marginal and para-marginal flaps are the most commonly used flap designs for germectomies, often combined with distal discharge. In both methods, the incision extends from the first molar to the second molar, while the discharge cut starts from the middle of the second molar, angling distally at 45° to reach the vestibular bone where the third molar germ is located. In the marginal technique, the flap design extends into the gingival sulcus and intra-papillar, while in the para-marginal technique, the incision is made between the attached gingiva and free gingiva [13].

The objective of this prospective study was to compare the effects of marginal and para-marginal flap designs in terms of wound healing, distal margin depth of the second molar, post-operative pain perception, maximum mouth opening, and swelling.

## 2. Materials and Methods

In this study, a total of forty patients, aged between 11 and 16 years (23 girls and 17 boys), were recruited. The average age of males was 14.6(±1.6) years old, while the average age of females was 13.91 (±1.82). These patients presented impacted third molar germs in either the right or left mandible, and extraction of these germs was recommended for prophylactic and orthodontic reasons. Prior to the surgical procedure, the parents of all patients received informed consent forms, which provided detailed information about the intervention, post-operative recommendations, and potential complications that may arise following the extractions. Both the patients and their parents were unaware of the specific incisional technique they would undergo (Supplementary Materials).

**Figure 1:**
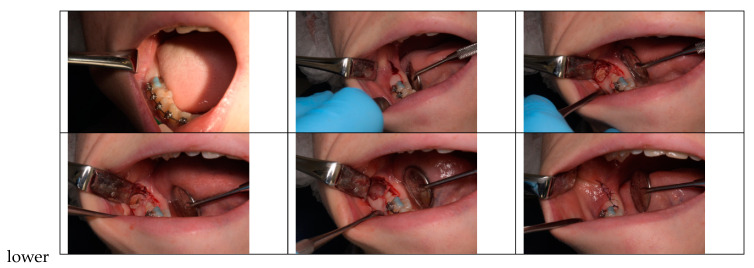
Marginal incision technique.

**Figure 2:**
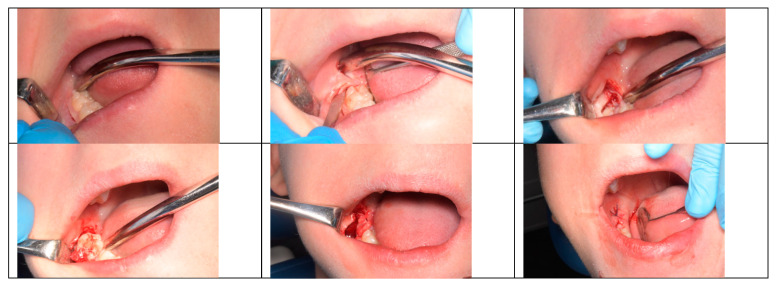
Para-marginal incision technique.

A total of forty germectomies were performed, with twenty involving the right mandibular third molars (RLM3) and twenty involving the left mandibular third molars (LLM3). Half of the germectomies were carried out using a marginal incision technique (Figure 1) (ten LLM3 and ten RLM3), while the other half utilized a para-marginal incision technique (Figure 2) (ten LLM3 and ten RLM3). The assignment of incisional techniques was randomized using the IBM SPSS^®^ computer program.

The surgical procedures were fully standardized, as all patients were operated on by a single trained operator. Prior to the surgery, a comprehensive evaluation of the orthopantomography was performed.

Before the surgical procedure, the following measurements were evaluated by a blinded and trained researcher, who was unaware of which patients would receive the marginal or para-marginal technique. The periodontal evaluation of the LM2 included the assessment of the plaque index (expressed as a percentage), bleeding on probing index (BoP), and probing depth (in millimeters) of the distal surface of the tooth using a periodontal probe. The maximum mouth opening was measured using a millimeter scale, from the incisal border of the upper incisor to the incisal border of the lower incisor. These measurements were recorded both before and after the surgical removal of the third molar and documented in a dataset.

Prior to the extraction, an inferior alveolar nerve block and a buccinator nerve block were administered.

One week after the surgery, a complete periodontal evaluation of the LM2 was conducted. Additionally, post-operative pain perception (assessed by the patient using a visual analog scale, VAS) and post-operative swelling were recorded. All measurements were collected and documented in the dataset, as previously specified.

The plaque index will be analyzed using a *t*-test, while the maximum mouth opening, distal probing, and post-operative pain will be evaluated using a mean test.

## 3. Results

For the present prospective study, forty patients, twenty-two females and eighteen males, between 11 and 16 y.o. were recruited. Forty germectomies were performed, half with marginal incision technique and half with para-marginal incision technique. No dropouts were reported.

### 3.1. Plaque Index

**Figure 3:**
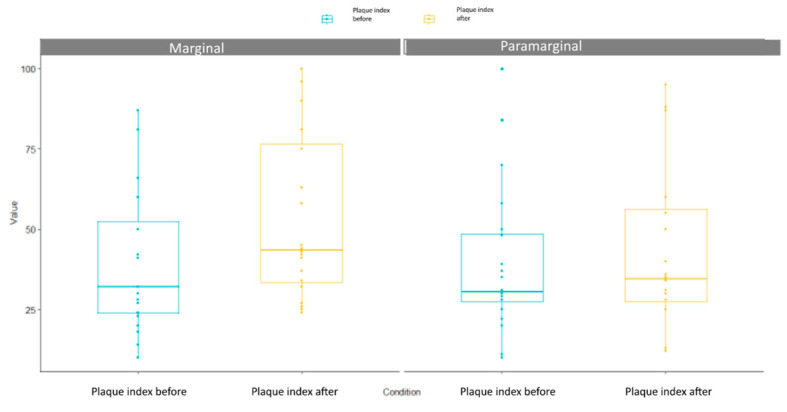
Plaque index box plot.

In Figure 3 and Table 1, it is appreciable that a higher percentage of plaque (>13%) was observed after performing the marginal flap, meanwhile a similar plaque percentage was reported before and after the para-marginal flap technique (>3.8%). SSD were reported between marginal flap technique compared with the para-marginal flap technique (*p*-value = 0.001264).

### 3.2. BoP Index

The pre-surgical BoP Index revealed that there was a slightly higher number of bleeding patients in the para-marginal group (nine patients, 45%) than in the marginal group (seven patients, 35%). Instead, no bleeding was reported in 13 patients (65%) belonging to the marginal group, and 11 patients (55%) in the para-marginal group. After marginal flap, no bleeding was reported in one patient (5%) and bleeding was experienced in the remaining nineteen patients (95%). For what concerns the para-marginal flap, BoP was reported in 6 patients (30%), and no bleeding was observed in 14 patients (70%). In general, there was a reduction in BoP of about 65% in para-marginal flap compared to the marginal one. For further clarification, please see Table 2.

### 3.3. Maximum Mouth Opening Index

**Figure 4:**
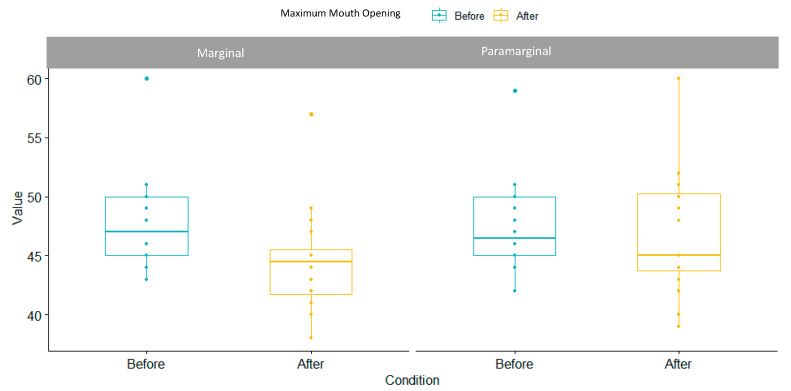
Maximum mouth opening box plot.

Maximum mouth opening range was similar between groups at baseline and it slightly diminished in marginal group (3.5%) compared to para-marginal group (0.7%), as specified in Table 3. In some cases, the maximum mouth opening in the para-marginal group was wider, as reported in Figure 4. SSD were reported between the two techniques, as the *p*-value was 0.001264.

### 3.4. Post-Operative Pain Perception

**Figure 5:**
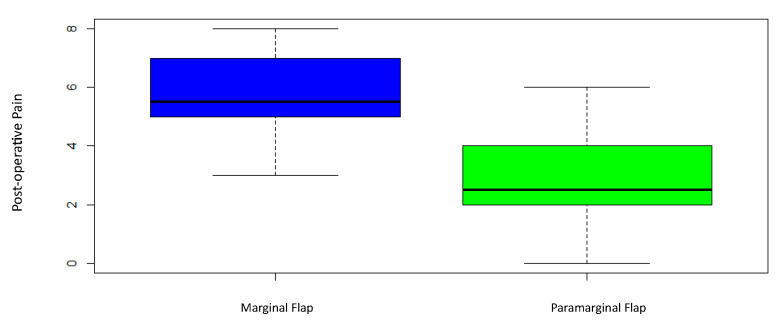
Post-pain perception box plot.

Highly SSD were found in patients receiving the para-marginal flap (5.75 ± 1.37), who reported a lower pain level, compared with the ones who received the marginal flap technique (2.95 ± 3.73), with a *p*-value of 0.000005 (Figure 5 and Table 4).

### 3.5. Post-Operative Swelling

All patients treated with a marginal flap experienced a visible swelling on the mid-cheek and molar region. A total of 11 patients (55%) receiving para-marginal flap did not experience any swelling, but 9 patients did (45%) (Table 5).

### 3.6. Distal Probing

**Figure 6:**
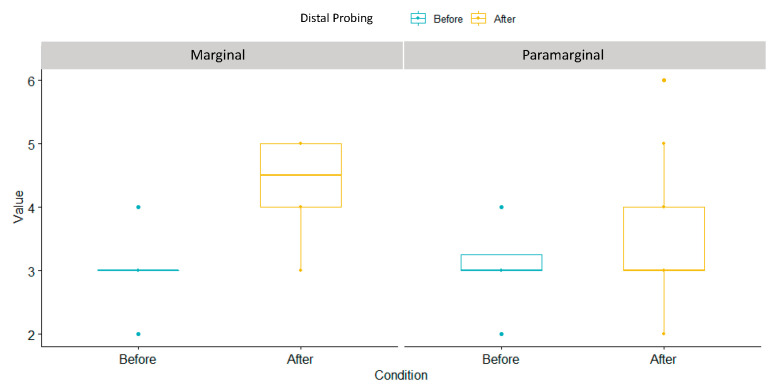
Distal probing box plot.

As reported in Figure 6 and Table 6, distal probing significantly increased in marginal incision (1.3 ± 0.47) in comparison with para-marginal incision (0.4 ± 0.687), as reported by the *p*-value = 0.0000275.

## 4. Discussion

Late germectomy of third mandibular molars represents a valuable alternative to early germectomy or delayed third molar extraction. Certainly, it is not risk-free, but it is undoubtedly the safest and minimally invasive alternative available at the present time. As previously mentioned, some well-known and frequently reported complications might occur. The right choice of the flap design represents one of the factors that might influence the occurrence or absence of post-operative complications. This is the main reason why the present study focused on two different flap designs: the marginal flap, which is mostly used for third molar extraction, and the para-marginal flap, which allows for the respect of the supracrestal tissue attachment and potentially preserves periodontal health.

According to our study, the marginal flap was more plaque-retentive, and a higher level of BoP on the distal aspect of the second lower molar was reported. In addition, patients reported greater difficulty in reaching the maximum mouth opening, which was diminished compared with the baseline. Post-operative pain perception, swelling, tumefaction, and distal probing were higher in those patients in which the marginal flap technique was carried out. Therefore, the results of the present study support the concept that the para-marginal approach is less invasive and more conservative in terms of periodontal health. From the patient’s perspective, the para-marginal technique was also better accepted, as this non-invasive incision leads to less post-operative discomfort in terms of maximum mouth opening, tumefaction, and pain perception.

Similar results were reported by Suarez Cunquiero et al.; in fact, in their study, a statistically significant increase in probing depth at the buccal and distal sites of the adjacent second molars was observed in the marginal flap group at day 5 and 10 after surgery. In the para-marginal flap group, a lower probing depth was always reported compared to the marginal flap group. On the other hand, similar results were reported in terms of plaque index, BoP, pain, trismus, and swelling for marginal and para-marginal approaches [14].

Conversely, Shahzad et al. reported that no statistically significant differences were found between the marginal flap and the para-marginal flap at week 1 and 2 in terms of wound dehiscence appearance (*p* > 0.05). Likewise, no significant differences were found in the buccal and distal probing depths of the adjacent second molar (*p* > 0.05) [15].

Again, Chalkoo et al., in a study carried out in 2015, showed that there were no significant differences between the marginal and para-marginal flaps in terms of maximum mouth opening before surgery, on the second and seventh day after surgery. However, both techniques were associated with a significant restricted mouth opening on the second day after surgery (*p* < 0.001) and a significant improvement at the seventh day after surgery (*p* < 0.001) [16].

Para-marginal and marginal incision techniques could also be used in highly aesthetic surgeries, for example, in canine surgeries. Kösger et al. found that there were no statistically significant differences between marginal and para-marginal flap designs in terms of plaque index, gingival index, and probing depth at any time point [17].

Since it is an anterior region, it is much easier to keep it clean and plaque-free, and, hence, it is likely that no appreciable differences in plaque retention were observed in this case. Conversely, in the area of third molars, there was a higher possibility of saliva stagnation, and the choice of flap might play a crucial role in achieving a more cleansable situation.

To avoid secondary effects after third molar surgery, many authors developed their own flap designs. Some of them are designed with a marginal approach, such as the envelope flap, the modified envelope flap, or the triangular flap. Other flap designs, such as the Szmyd flap, the modified Szmyd flap, and the modified triangular flap, all share the submarginal incision technique, which is comparable to a para-marginal approach [18].

According to Chen et al., the para-marginal flap showed greater periodontal depth reduction compared to other types of flaps that adopt the marginal incision. This can be explained by the fact that the sub-marginal incision flaps maintain gingival margin height by eliminating the need to detach the keratinized gingiva, leaving a complete gingival collar around the adjacent molar. This procedure minimizes the impact of surgery on the periodontal health of the adjacent second molar [19]. Moreover, the apical incision allows for better papillae conservation, promoting higher standards of oral hygiene and reducing plaque retention. A lower proliferation of bacteria due to stagnation and inflammation leads to a lower BoP level, as observed in our study in the para-marginal group.

Furthermore, it should be considered that in the absence of inflammation, faster and better wound healing is promoted. In a study by Jakse et al., 57% of cases treated with a sulcular flap approach resulted in wound healing failure or dehiscence. In contrast, only 10% of cases approached with a sub-marginal flap developed wound dehiscence after third molar surgery [20]. Although no cases of wound healing failure were reported in our study within the one-week post-operative period, a higher level of BoP was detected in the marginal flap group, suggesting an increased risk of wound healing failure. BoP and wound healing failure can also occur when there is a lack of flap stability and increased tension on the surgical site. Some authors argue that more invasive procedures, such as para-marginal approaches with vertical incisions, result in a greater probability of experiencing higher levels of pain. It is believed that a more conservative approach decreases post-operative pain and tumefaction [21,22].

Furthermore, it is important to note that pain, tumefaction, and restricted mouth opening were significantly influenced by the osteotomy and surgery time, which, in turn, were greatly impacted by the position and inclination of the third molars in relation to the mandibular ramus. These factors can be assessed using classifications such as Pell and Gregory’s and Winter’s classifications. According to these classifications, the more favorable the position and inclination of the third molars, the less post-operative discomfort is experienced.

Considering these factors, it was evident that undergoing germectomy, the removal of third molars at an earlier stage, is highly recommended instead of waiting for complete formation. This recommendation is supported by the fact that the final position of the third molars cannot be accurately predicted [23].

By opting for germectomy procedures at an earlier stage, the surgical procedures can be completed in a shorter duration, thereby reducing post-operative discomfort. This shorter surgical time not only contributes to a decrease in pain, tumefaction, and limited mouth opening but also provides an additional rationale for recommending germectomy over waiting for the complete formation of third molars [24].

The unpredictability of the final position of the third molars further reinforces the importance of germectomy as a proactive approach. Waiting for complete formation may lead to complications and difficulties during the surgical procedure, resulting in increased pain and discomfort for the patient. Thus, the proactive approach of germectomy offers a more controlled and predictable outcome, leading to improved patient outcomes and satisfaction [25].

In summary, pain, tumefaction, and restricted mouth opening following third molar surgery are closely associated with the osteotomy and surgery time, which are influenced by the position and inclination of the molars. To minimize post-operative discomfort, it is advisable to opt for germectomy procedures at an earlier stage, rather than waiting for complete formation, due to the uncertainty regarding the final position of the molars. This proactive approach ensures shorter surgical procedures and enhances predictability, ultimately benefiting the patient’s well-being and satisfaction [26].

Various solutions were investigated to reduce post-operative discomfort, such as the application of cryotherapy and laser therapy, but only minimal improvements were detected. Currently, time and medications remain the primary solutions for reducing post-surgical complications [27,28].

Furthermore, the formation of a periodontal pocket distal to the adjacent second molar is the most common and well-known post-operative complication, as described by many authors. Although a direct correlation between pain, tumefaction, probing depths, and bleeding on probing was not established, it appears that younger patients are less prone to develop periodontal pockets, and generally, the probing depths are significantly lower compared to adult patients [19,20,21,22,29]. In our study, a higher probing depth was observed in patients treated with a marginal approach. However, age seemed to play a role in the development of a periodontal pocket distal to the second molar. This is influenced by the position of the third molar itself, as well as the difficulty in maintaining oral hygiene in the retromolar area [30].

In conclusion, based on our study comparing the marginal and para-marginal flap techniques for late germectomy of third mandibular molars, it was evident that the para-marginal approach presents several advantages, making it a safer and more conservative alternative in terms of periodontal health [31]. By utilizing the para-marginal incision technique, the incidence of plaque retention was significantly reduced, resulting in lower levels of bleeding on probing (BoP). Additionally, patients experienced less post-operative discomfort, including pain, swelling, and limited mouth opening.

The preservation of periodontal health achieved through the para-marginal flap technique not only contributed to improved patient acceptance but also facilitated faster and superior wound healing. These benefits collectively established the para-marginal approach as a valuable option in late germectomy procedures [19]. Nonetheless, further long-term studies are necessary to validate these findings and gain a comprehensive understanding of the impact on long-term periodontal health.

The potential implications of our research are noteworthy, as they highlight the significance of adopting the para-marginal flap technique in dental practice. By prioritizing periodontal health and patient comfort, dental professionals can enhance the overall success and outcomes of late germectomy procedures. Consequently, incorporating the para-marginal approach into clinical protocols may lead to a paradigm shift in the field, ensuring optimal patient care and satisfaction.

Furthermore, comprehensive investigations are warranted to evaluate the long-term effects of the para-marginal technique on periodontal health. Longitudinal studies assessing factors such as probing depths, attachment loss, and alveolar bone levels will provide valuable insights into the extended benefits and sustainability of this approach. By elucidating the impact on long-term periodontal health, dental practitioners can make informed decisions and provide evidence-based recommendations to their patients.

In summary, our study supports the para-marginal flap technique as a safer and more conservative option for late germectomy procedures of third mandibular molars. The observed advantages, including reduced plaque retention, lower BoP levels, and decreased post-operative discomfort, emphasize the importance of embracing this approach to promote periodontal health and improve patient outcomes. Continued research and clinical validation will refine our understanding and establish the long-term benefits of the para-marginal flap technique in dental practice.

## 5. Conclusions

In light of the above, the para-marginal flap design showed more promising results compared to the marginal flap design. Periodontal health is better preserved in terms of PI, PD and BoP, and MOM, PP and PT are reduced in the para-marginal flap compared with the marginal flap, which represents less discomfort and consequences for the patient’s prospective. Therefore, we recommend to have a sub-marginal approach in late germectomies, as secondary effects seem to be strongly reduced, even if further investigations on this topic are highly suggested.

## Figures and Tables

**Table 1:** Descriptive measurements of the plaque index values.

Flap Design	Min	Median	Mean	Variance	Standar Deviation	Max	*p*-Value
Marginal	2	12.5	13.3	106	10.3	49	0.001264
Paramarginal	−5	3	3.85	35.6	5.97	18	

**Table 2:** Descriptive measurements of the BoP Index pre and post-surgery.

Pre-Surgical BoP	Flap Design	n	prop
No bleeding	Marginal	13	65%
No bleeding	Paramarginal	11	55%
Bleeding	Marginal	7	35%
Bleeding	Paramarginal	9	45%
**Post-surgical Bop**	**Flap design**	**n**	**prop**
No bleeding	Marginal	1	5%
No bleeding	Paramarginal	14	70%
Bleeding	Marginal	19	95%
Bleeding	Paramarginal	6	30%

**Table 3:** Maximum mouth opening values.

Flap Design	Min	Median	Mean	Variance	Standar Deviation	Max	*p*-Value
Marginal	−8	−3	−3.5	3.32	1.82	0	0.001264
Paramarginal	−6	−0.5	−0.7	4.75	2.18	3	

**Table 4:** Descriptive measurements of the post-operative pain perception.

Flap Design	Min	Median	Mean	Variance	Standar Deviation	Max	*p*-Value
Marginal	3	5.5	5.75	1.88	1.37	8	0.000005
Paramarginal	0	2.5	2.95	3.73	1.93	6	

**Table 5:** Descriptive measurements of the post-operative swelling.

Post-Operative Swelling	Flap Design	n	prop
No	Paramarginal	11	55%
Yes	Paramarginal	9	45%
Yes	Marginal	20	100%

**Table 6:** Descriptive measurements of the distal probing.

Flap Design	Min	Median	Mean	Variance	Standar Deviation	Max	*p*-Value
Marginal	1	1	1.3	0.221	0.47	2	0.0000275
Paramarginal	−1	0	0.4	0.463	0.681	2	

## Data Availability

Not applicable.

## Supplementary Materials

The following supporting information can be downloaded at: www.mdpi.com/xxx/s1.
